# The effect of GLP-1 receptor agonists on renal outcomes: a systematic review and meta-analysis

**DOI:** 10.1093/ndt/gfaf193

**Published:** 2025-09-22

**Authors:** Takaya Sasaki, Samantha My-Linh Giang, Jiajia Wu, Takashi Yokoo, Martin Gallagher, Rinaldo Bellomo, Amanda Ying Wang

**Affiliations:** Renal and Metabolic Division, George Institute for Global Health, University of New South Wales, Sydney, NSW, Australia; Division of Nephrology and Hypertension, Department of Internal Medicine, The Jikei University School of Medicine, Tokyo, Japan; Department of Nephrology, Concord Repatriation General Hospital, Sydney, NSW, Australia; Concord Clinical School, Faculty of Medicine, University of Sydney, Sydney, NSW, Australia; Department of Nephrology, Sir Run Run Shaw Hospital, Zhejiang University, Hangzhou, China; Division of Nephrology and Hypertension, Department of Internal Medicine, The Jikei University School of Medicine, Tokyo, Japan; Renal and Metabolic Division, George Institute for Global Health, University of New South Wales, Sydney, NSW, Australia; Australian and New Zealand Intensive Care Research Centre (ANZIC-RC), School of Public Health and Preventative Medicine, Monash University, Melbourne, Australia; Renal and Metabolic Division, George Institute for Global Health, University of New South Wales, Sydney, NSW, Australia; Concord Clinical School, Faculty of Medicine, University of Sydney, Sydney, NSW, Australia; The Faculty of Medicine and Health Sciences, Macquarie University, Sydney, NSW, Australia

**Keywords:** chronic kidney disease, glucagon-like peptide-1 receptor agonists, systematic review

## Abstract

**Background and hypothesis:**

Glucagon-like peptide-1 receptor agonists (GLP1-RAs) are anti-hyperglycaemic agents, with cardioprotective effects, however their renal protective effects remain unclear. We aimed to assess the effects of GLP1-RAs on renal outcomes in patients with or without diabetes.

**Methods:**

We performed a systematic review and meta-analysis with Medline, EMBASE and the Cochrane Register searched to December 2024. The primary outcome was the composite of kidney failure (defined as estimated glomerular filtration rate <15 mL/min/1.73 m^2^) or dialysis requirement, worsening of renal function and changes in proteinuria. Subgroup analysis was performed based on diabetic status, a (CKD) and individual GLP1-RA drugs. Relative risks (RR) with 95% confidence intervals (CI) for individual trials were pooled using random effects models.

**Results:**

We identified 19 trials including 90 882 patients. Mean age was 60.8 years and mean follow-up was 25.9 months. GLP1-RAs were associated with a 19% reduction in the risk of primary renal outcome (RR 0.81, 95% CI 0.73–0.89), a 12% reduction in renal functional decline (RR 0.88, 95% CI 0.81–0.95) and a 0.45 mL/min/1.73 m^2^ reduction in yearly loss [16 trials, mean difference (MD) 0.45, 95% CI 0.10–0.81]. GLP1-RAs also reduced microalbuminuria by 24% (RR 0.76, 95% CI 0.71–0.82), HbA1c (units: %) by 0.61 (MD –0.61, 95% CI –0.76 to –0.49) and body weight by 5 kg (MD –5.24, 95% CI –7.46 to –3.02). Although there were no significant differences in progression to kidney failure (RR 0.86, 95% CI 0.71–1.05), GLP1-RAs reduced the incidence of major adverse cardiovascular events by 15% (RR 0.85, 95% CI 0.81–0.90) and all-cause mortality by 14% (RR 0.86, 95% CI 0.82–0.91). No significant differences were seen in severe adverse events (RR 0.96, 95% CI 0.90–1.01). However, there were more gastroenterological side effects.

**Conclusions:**

GLP1-RAs demonstrated cardiovascular and renal benefits. Further high-quality randomized trials assessing their effects in patients without diabetes, with or without proteinuria and/or CKD are needed.

## Graphical Abstract

**Graphical Abstract:**
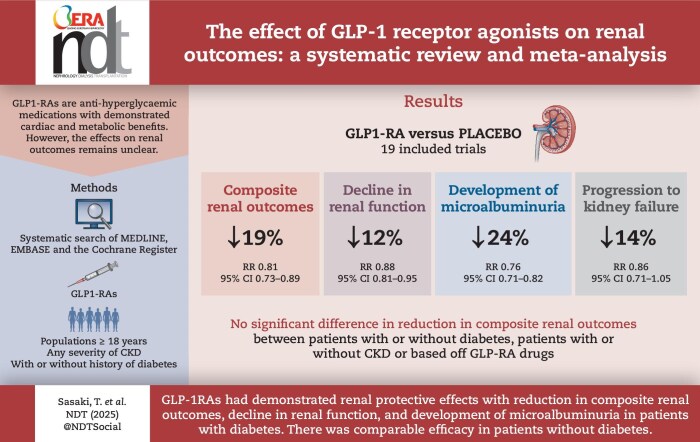

KEY LEARNING POINTS **What was known:**

- Glucagon-like peptide-1 receptor agonists (GLP-1RAs) have established cardiovascular benefits and glycaemic control effects in patients with type 2 diabetes.
- Evidence regarding the effect of GLP-1RAs on renal outcomes has been inconsistent across individual clinical trials.
- There was paucity of data on the effect of GLP-1RAs on kidney outcomes in the non-diabetic population.

**This study adds:**

- GLP-1RAs significantly reduce the risk of composite renal outcomes, decline in kidney function and progression of albuminuria.
- Cardiovascular and survival benefits of GLP-1RAs are reaffirmed across a broad population, with an acceptable safety profile.
- These benefits appear to extend beyond glycaemic control, suggesting potential renal utility in non-diabetic patients.

**Potential impact:**

- Clinicians may consider GLP-1RAs as a dual-purpose therapeutic agent for both cardiovascular and renal protection.
- Findings support the design of future trials focusing on GLP-1RAs in non-diabetic chronic kidney disease populations.
- Results may influence clinical guidelines to expand the indications of GLP-1RAs beyond glycaemic control in diabetes.

## INTRODUCTION

Chronic kidney disease (CKD) contributes significantly to morbidity and mortality rates, acting as a direct cause and as a significant cardiovascular risk factor. Diabetes, hypertension and cardiovascular disease are among the most prevalent risk factors for CKD [1]. The global burden of CKD continues to grow, with 9.1% of the population being affected at various stages of the disease and resulting in 1.2 million deaths in 2017 [2].

Glucagon-like peptide-1 receptor agonists (GLP1-RAs) are a class of anti-hyperglycaemic agents, initially developed for

management of patients with type 2 diabetes [3]. In addition to their role in glycaemic control, current literature has highlighted their cardioprotective effects in the diabetic population [4–6]. More recently, studies have suggested a possible beneficial effects on renal outcomes [6].

The putative renal protective effects of GLP1-RAs are poorly understood and may include body weight reduction and glycaemic control, as well as direct effects on kidney function [7]. Despite multiple studies investigating these properties, however, there is often exclusion of patients with advanced CKD, further complicating interpretation of the renal effects in GLP1-RAs [8]. Furthermore, with differing trial designs and patient populations, their effects on renal outcomes remain inconsistent [6].

We therefore performed a systematic review and meta-analysis to assess the effects of GLP1-RAs on renal outcomes in patients with or without diabetes, obesity and CKD.

## MATERIALS AND METHODS

### Search strategies

We conducted the systematic review following PRISMA (Preferred Reporting Items for Systematic reviews and Meta-Analyses) 2020 statement [9]. The following databases were searched including MEDLINE via OvidSP (from 1946 to 10 December 2024), EMBASE via OvidSP (from 1946 to 10 December 2024) and the Cochrane Central Register of Controlled Trials (from 1946 to 10 December 2024). Further studies were obtained via hand searches of eligible studies and reviewing reference lists of relevant studies, selected conference abstracts and via ClinicalTrials.gov. website. There were no language or publication period restrictions. Detailed search strategies are provided in Supplementary data, Table S1.

### Study selection

We included randomized controlled trials (RCTs) that reported the impact of GLP1-RAs on kidney-related outcomes as defined below. The exclusion criteria were as follows: (i) studies in paediatric population, (ii) studies in the population with specific comorbidities (e.g. liver cirrhosis), (iii) clinical trials involving fewer than 100 participants and (iv) clinical trials with study period of ≤12 weeks. Papers were independently assessed for inclusion by two reviewers (T.S. and J.J.W.). Discrepancies between reviewers on eligibility of a study were resolved by a third reviewer (S.M.G.) and by consensus discussion.

### Study outcomes

The primary outcome was a composite of renal outcomes defined as development of kidney failure [defined as estimated glomerular filtration rate (eGFR) <15 mL/min/1.73 m^2^] or dialysis requirement, worsening of renal function (as defined by the authors) and changes in proteinuria. Secondary outcomes were kidney-related outcomes, namely development of kidney failure, substantial loss of kidney function, slope of eGFR, change in urinary albuminuria excretion and new onset of macroalbuminuria. Tertiary outcomes included major adverse cardiovascular events (MACE) and all-cause mortality. Also, other outcomes included changes in metabolic parameters such as glycated haemoglobin (HbA1c), body weight and adverse outcomes. Pre-specific subgroup analysis was determined by the presence or absence of diabetes, presence of CKD (defined as eGFR <60 mL/min/1.73 m^2^ or by study definition) and by GLP1-RA drug.

### Data extraction and quality assessment

Two authors (T.S. and J.J.W.) independently extracted data using a standard data extraction sheet. Data extracted from each study included baseline characteristics of study participants, intervention, duration of follow-up and outcomes. Risk of bias for RCTs was independently assessed by two reviewers (T.S. and J.J.W.) using the risk-of-bias tool version 2 (RoB2). Discrepancies between reviewers on the extracted data and the risk of bias in studies were resolved by other investigators (S.M.G. and A.Y.W.) and by consensus discussion. In studies involving multi-arms, we pre-specifically combined all control arms into one group, in comparison with GLP1-RAs.

### Data synthesis and analysis

Pooled analyses using random-effect model were performed to estimate relative risks (RRs) and 95% confidence intervals (CIs) for dichotomous variables, and mean differences (MDs) or standard mean differences and 95% CIs calculated for continuous variables. Statistical heterogeneities were assessed using *I*^2^ and Cochran Q test. An *I*^2^ of <25% represents low, *I*^2^ of 25%–75% represents moderate and *I*^2^ of >75% represents high heterogeneity [10]. A prespecified subgroup analysis according to with or without diabetes was performed to explore sources of heterogeneity. Value *P* < .05 was considered statistically significant for all analyses. All the analyses were conducted using R version 4.2.2 with ‘meta’ package.

## RESULTS

### Baseline characteristics

**Figure 1:**
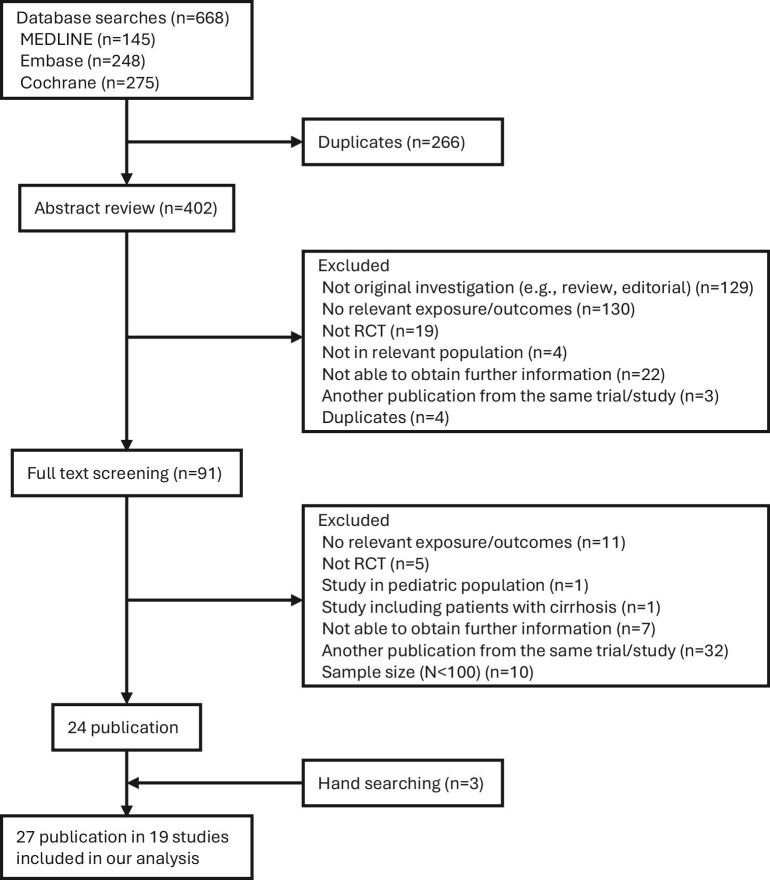
The PRISMA flow program. The PRISMA flow diagram outlines the study selection process for this systematic review. The diagram details the number of records identified through database searches, the number of duplicates removed, the records screened, the number of full-text articles assessed for eligibility and the final number of studies included in the analysis. Reasons for exclusion at each stage are documented.

Our literature search yielded 19 trials [4, 6, 11–27], inclusive of 90 882 randomly assigned patients (46 693 patients in the intervention group and 46 461 patients in the control group) with a mean overall follow-up of 26 months (ranging from 6 to 72 months) (Fig. 1). The baseline characteristics of each study are summarized in Table 1. The study populations were male predominant (57%, 51 802 patients) and mean age was 61 years. All studies were prospective randomized clinical trials. Study size ranged between 159 and 17 604 patients. Amongst the included trials, 12 trials including 66 053 patients primarily assessed those with type 2 diabetes, 4 trials assessed 20 277 patients with obesity, 2 trials assessed 1369 patients with both type 2 diabetes and obesity, and 1 trial assessed 3183 patients with high cardiovascular risk factors. Of the 252 items found (18 trials and 14 outcomes), 23 contained discrepancies and agreement was 90.90%. Kappa was not calculated as the data were not categorical in a manner suitable for kappa statistics.

**Table 1:** Baseline characteristics of the included studies.

Study name	Inclusion criteria	Intervention, number of participants	Control, number of participants	Intervention	Dose	Control	Male sex (%)	Age, years	Diabetes mellitus (%)	BMI (kg/m²)	Baseline kidney function (mL/min/1.73 m²)	Baseline proteinuria	Follow-up (months)
AWARD7	Adults with T2DM and moderate to severe CKD	576	194	Dulaglutide	0.75, 1.5 mg	Insulin glargine	52.2	64.6	100	32.5	38.3	214.2 mg/g (mean of the median for each arm)	12
EXCEL	Adults with T2DM	14 752	7396	Exenatide	2 mg	Placebo	62	62	100	32.7	76.3	NA^a^	38.4
SUSTAIN6	Adults with T2DM and with high CV risk	3297	1649	Semaglutide	0.5, 1.0 mg	Placebo	64.6	60.7	100	32.8	76.1	38.6 mg/g	25.1
PIONEER6	Adults with high CV risk	3183	1592	Semaglutide	14 mg (target)	Placebo	66	68.4	100	32.3	74	NA	15.9
LIRA-RENAL	Adults with T2DM	277	137	Liraglutide	1.8 mg	Placebo	50.5	67.1	100	34.0	45.4 (mean of gm^b^)	62.6 mg/g (mean of gm)	6
HARMONY	Adults with T2DM and CV disease	9463	4732	Albiglutide	30 mg	Placebo	69.4	64.2	100	32.3	79	NA	19.2
REWIND	Adults with T2DM and high CV risk	9901	4952	Dulaglutide	1.5 mg	Placebo	53.7	66.2	100	32.3	75	1.84 mg/g	72
AMPLITUDE-O	Adults with T2DM with high CV risk or CKD	4076	1359	Efpeglenatide	4, 6 mg	Placebo	64.5	67	100	32.7	72.4	28.3 mg/g median	21.7
SURPASS4	Adults with T2DM, high BMI and high CV risk	1995	1000	Tirzepatide	5 mg, 10 mg, or 15 mg	Insulin glargine	62	63.6	100	32.6	81.3	15.0 mg/g median	12
STEP 1	Adults with high BMI without diabetes	1961	655	Semaglutide	2.4 mg	Placebo	25.9	46.3	0	37.9	76.3	NA	15.6
STEP 2	Adults with high BMI without diabetes	1210	403	Semaglutide	1.0, 2.4 mg	Placebo	49.1	55	100	35.7	93.3	13.1 mg/g mean of gm	15.6
STEP 3	Adults with high BMI without diabetes	611	204	Semaglutide	2.4 mg	Placebo	19	46	0	38	96.6	NA	15.6
ChiCTR-1701082	Adults with T2DM and high BMI	159	80	Exenatide	2 mg	Insulin glargine	54.7	48.7	100	27.3	NA	80 mg/L median	5.5
LEADER	Adults with T2DM and high CV risk	9340	4672	Liraglutide	1.8 mg	Placebo	64.3	64.3	100	32.5	80	Macroalbuminuria 10.4%	46
ELIXA	Adults with T2DM and high CV risk	6068	3034	Lixisenatide	20 microgram (maximum)	Placebo	60.3	69.4	100	30.2	76	10.3 mg/g (mean of median)	24.9
GRADE	Adults with T2DM	5047	3785	Liraglutide	1.8 mg (maximum)	Glargine, glimepiride, or sitagliptin	63.6	57.2	100	34.3	94.9	6.4 mg/g (median)	60
FLOW	Adults with T2DM and CKD	3533	1766	Semaglutide	1.0 mg	Placebo	69.7	66.6	100	32	47	567.6 mg/g (median)	40.8
SELECT	Adults with high CV risk and high BMI with no history of T2DM	17 604	8801	Semaglutide	2.4 mg	Placebo	72.4	61.6	0	33.3	82.4	7.4 mg/g (median)	39.8
SMART	Adults with CKD and high BMI	101	50	Semaglutide	2.4 mg	Placebo	60.4	55.8	0	36.2	65	251 mg/g (median)	6

BMI, body mass index; CV, cardiovascular; T2DM, type 2 diabetes mellitus.

^a^Not available.

^b^Geometric mean.

Semaglutide was used for the majority of the trials (8 trials, 16 380 patients) with doses ranging between 1 mg and 14 mg. Other interventions included liraglutide (3 trials, 6070 patients), exenatide (2 trials, 7435 patients), dulaglutide (2 trials, 5331 patients), albiglutide (1 trial, 4731 patients), efpeglenatide (1 trial, 2717 patients), lixisenatide (1 trial, 3034 patients) and tirzepatide (1 trial, 995 patients). The majority of the trials used placebo as their control group (15 trials, 42 994 patients). Insulin glargine was also used as a control (3 trials, 1274 patients) and the GRADE trial used glargine, glimepiride or sitagliptin as the control.

### Risk of bias

The risk of bias for each randomized controlled trial is presented in Supplementary data, Figs S1–S15. All trials used a random method to generate the allocation sequence. Blinding of participants and personnel was judged to be at low risk in all trials. Similarly, all trials were considered at low risk for deviations from the intended interventions and at low risk for incomplete outcome reporting. Majority of trials were considered low risk for measurement of outcomes, except for AWARD 7 (Dulaglutide versus insulin glargine in patients with type 2 diabetes and moderate-to-severe chronic kidney disease) SURPASS 4 (Tirzepatide versus insulin glargine in type 2 diabetes and increased cardiovascular risk) and GRADE (Glycemia Reduction Approaches in Type 2 Diabetes: A Comparative Effectiveness). The majority of trials were considered low risk of selective reporting, except for SUSTAIN 6 (Semaglutide and Cardiovascular Outcomes in Patients with Type 2 Diabetes) PIONEER 6 (Oral Semaglutide and Cardiovascular Outcomes in Patients with Type 2 Diabetes) SURPASS 4, STEP 1 (Once-Weekly Semaglutide in Adults with Overweight or Obesity) STEP 2 (Semaglutide 2·4 mg once a week in adults with overweight or obesity, and type 2 diabetes) STEP 3 (Effect of Subcutaneous Semaglutide vs Placebo as an Adjunct to Intensive Behavioral Therapy on Body Weight in Adults With Overweight or Obesity) ELIXA (Lixisenatide in Patients with Type 2 Diabetes and Acute Coronary Syndrome) and SELECT (Semaglutide and Cardiovascular Outcomes in Obesity without Diabetes) which were assessed as some concern. Agreement for assessor’s overall judgement was 90.91% with weighted kappa of 0.76, indicating substantial agreement.

### Primary outcomes

**Figure 2:**
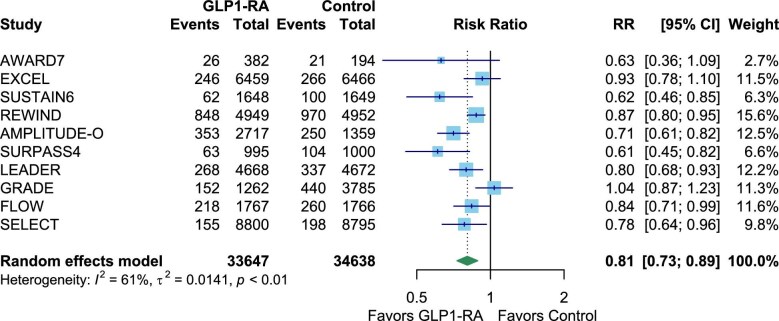
The effect of GLP1-RAs on kidney composite outcomes. Kidney composite outcomes are defined as combined renal outcomes including kidney dysfunction as defined in each individual study.

**Figure 3:**
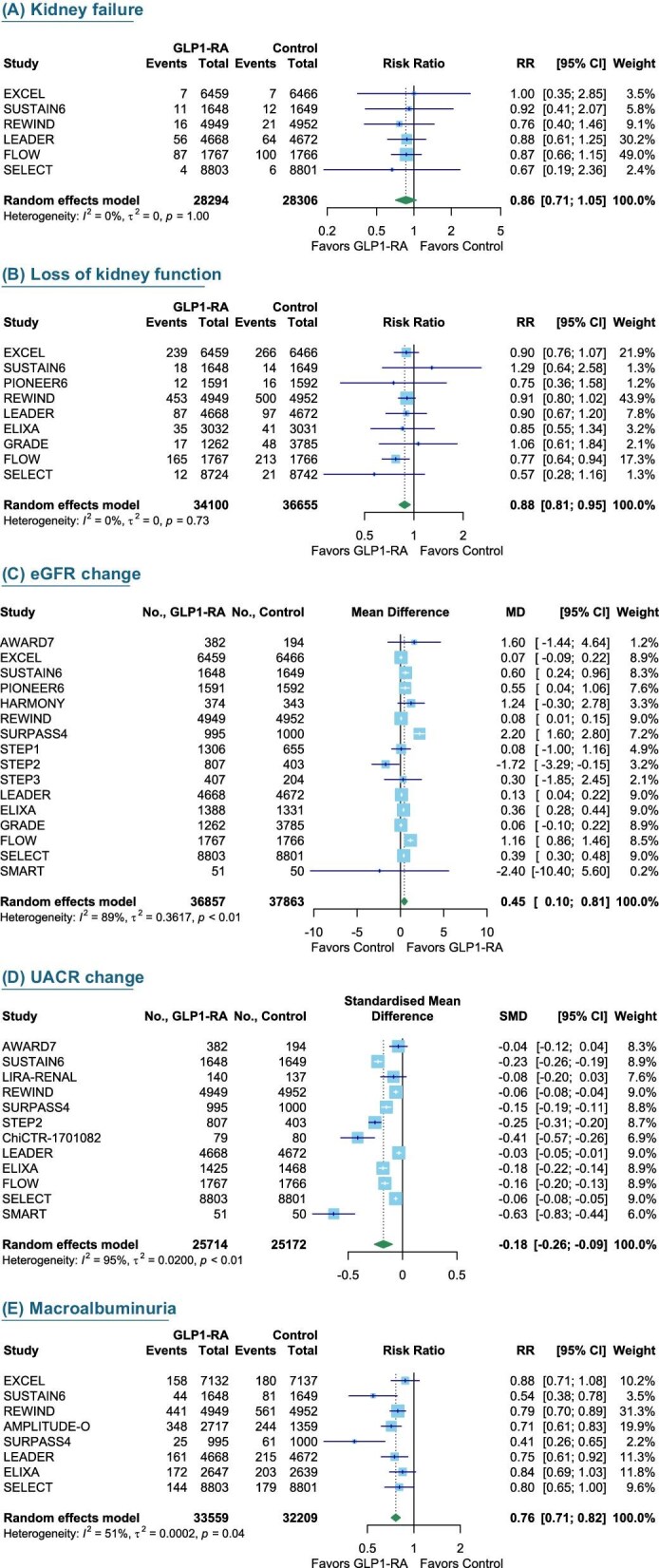
The effect of GLP1-RAs on individual kidney-related outcomes. Individual kidney outcomes are as follows: incidence of kidney failure (**A**), incidence of loss of kidney function (**B**), annual change in eGFR (**C**), changes in albuminuria between the baseline and last visits (**D**) and incidence of macroalbuminuria (**E**). RR, risk ratio; UACR, urine albumin–creatinine ratio.

**Figure 4:**
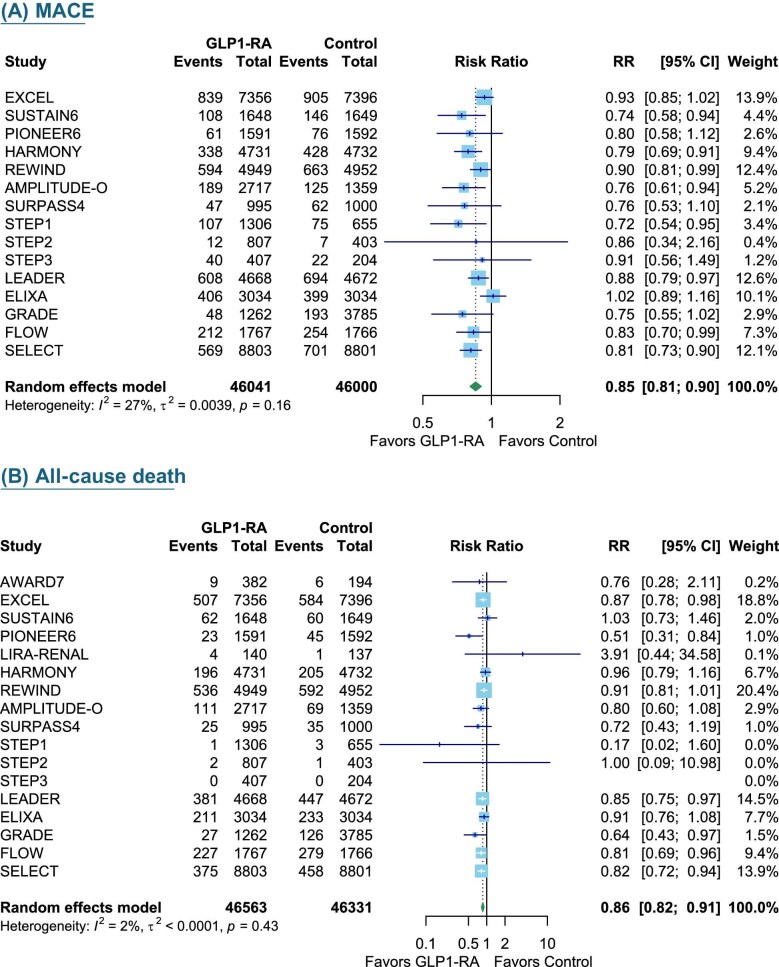
The effect of GLP1-RAs on MACE and all-cause mortality. MACE (**A**) and all-cause mortality (**B**). RR, risk ratio.

Compared with control interventions, GLP1-RAs significantly reduced risk of the primary outcome (composite renal outcomes) by 19% (10 trials, 33 647 participants; RR 0.81, 95% CI 0.73–0.89, *I*^2^ = 61%) Fig. 2. The risk of decline in renal function was reduced by 12% (9 trials, 34 100 participants; RR 0.88, 95% CI 0.81–0.95, *I*^2^ = 0%). The annual decline in eGFR was also reduced, with an MD of 0.45 mL/min/1.73 m^2^ per year (16 trials, 30 538 participants; MD 0.45, 95% CI 0.10–0.81, *I*^2^ = 89%). Additionally, GLP1-RAs reduced the risk of developing microalbuminuria by 24% (10 trials, 16 720 participants; RR 0.76, 95% CI 0.71–0.82, *I^2^* = 95%) (Fig. 3). There was, however, no significant difference in progression to kidney failure (6 trials, 28 294 participants; RR 0.86, CI 0.71–1.05, *I*^2^ = 95%) (Fig. 4).

Subgroup analysis showed a similar effect on the primary outcome in patients with diabetes (9 trials, 24 847 patients; RR 0.80, 95% CI 0.72–0.90, *I*^2^ = 65%) compared with patients without diabetes (1 trial, 8800 patients; RR 0.81, 95% CI 0.72–0.89, *I*^2^ = 61%) (Supplementary data, Fig. S18). Further subgroup analysis similarly showed comparable reduction in renal failure, loss of kidney function, reduction in eGFR decline and change in microalbuminuria.

Subgroup analysis also demonstrated a similar effect on composite renal outcomes in patients with chronic kidney disease (9 trials, 15 258 patients; RR 0.80, 95% CI 0.72–0.89, *I*^2^ = 50%) compared with patients with no history of chronic kidney disease (6 trials, 17 769 patients; RR 0.75, 95% CI 0.63–0.89, *I*^2^ = 33%) (Supplementary data, Fig. S20).

Further subgroup analysis revealed reduced risk of composite renal outcomes in majority of GLP1-RA drug classes, including semaglutide (3 trials, 12 215 patients; RR 0.78, 95% CI 0.68–0.89, *I*^2^ = 29%), efpeglenatide (1 trial, 2717 patients; RR 0.71, 95% CI 0.61–0.82) and tirzepatide (1 trial, 995 patients; RR 0.61, 95% CI 0.45–0.82). There was no statistically significant difference for dulaglutide (2 trials, 5331 patients; RR 0.83, 95% CI 0.66–1.05, *I*^2^ = 27%), exenatide (1 trial, 6459 patients, 95% CI 0.70–1.10) and liraglutide (2 trials, 5930 patients; RR 0.91, 95% CI 0.70–1.17, *I*^2^ = 80%) (Supplementary data, Fig. S22).

### Glycaemic control and weight reduction

GLP1-RAs improved glycaemic control and weight reduction. HbA1c (units: %) was reduced by 0.61 (15 trials, 20 512 participants; MD –0.61, 95% CI –0.76 to –0.49, *I*^2^ = 98%) and average weight loss was approximately 5 kg (15 trials, 27 905 participants; MD –5.24 kg, 95% CI –7.46 to –3.02, *I*^2^ = 98%) (Supplementary data, Fig. S16).

Subgroup analysis demonstrated a similar reduction in HbA1c in patients with diabetes (12 trials, 24 769 patients; RR –0.69, 95% CI –0.86 to –0.53, *I*^2^ = 96%) compared with patients without diabetes (3 trials, 1764 patients; RR –0.61, 95% CI –0.76 to –0.46, *I*^2^ = 98%).

### Cardiovascular outcomes

GLP1-RAs demonstrated cardiac protective effects and survival benefits with a 15% reduction in MACE (15 trials, 46 041 participants; RR 0.85, 95% CI 0.81–0.90, *I*^2^ = 27%) and a 14% reduction in all-cause mortality (17 trials, 46 563 participants; RR 0.86, 95% CI 0.81–0.91, *I*^2^ = 2%) (Fig. 4).

Subgroup analysis revealed similar reduction in MACE between patients with diabetes (12 trials, 35 525 patients; RR 0.86, 95% CI 0.81–0.92, *I*^2^ = 28%) and patients without diabetes (3 trials, 10 516 patients; RR 0.80, 95% CI 0.73–0.89, *I*^2^ = 27%).

### Safety outcomes

The safety profile of GLP1-RAs was generally favourable. There was no significant increase in the incidence of overall severe adverse events (18 trials, 46 611 participants; RR 0.96, 95% CI 0.90–1.01, *I*^2^ = 79%), acute kidney injury (12 trials, 36 568 participants; RR 0.94, 95% CI 0.86–1.04, *I*^2^ = 0%), or hypoglycemia (16 trials, 37 757 participants; RR 0.89, 95% CI 0.66–1.20, *I*^2^ = 85%). However, gastrointestinal side effects (18 trials, 46 611 participants; RR 1.75, 95% CI 1.36–2.25, *I*^2^ = 94%) were more common in patients who received GLP1-RAs compared with placebo (Supplementary data, Fig. S17).

Subgroup analysis revealed greater likelihood of overall severe adverse events (SAE) such as pancreatitis in patients with no diabetes (4 trials, 46 611 participants; RR 0.96, 95% CI 0.90–1.01, *I*^2^ = 82%) compared with patients with diabetes (14 trials, 36 044 participants; RR 0.94, 95% CI 0.89–1.00, *I*^2^ = 79%) (Supplementary data, Fig. S19). There was otherwise no demonstrated difference between patients with and without diabetes in the risk ratio of severe hypoglycaemia and acute kidney injury. There was a similar increased risk of gastrointestinal symptoms in both patients with diabetes (14 trials, 36 044 participants; RR 1.86, 95% CI 1.36–2.55, *I*^2^ = 95%) and without diabetes subgroups (4 trials, 10 567 participants; RR 1.36, 95% CI 1.10–1.69, *I*^2^ = 86%).

Further subgroup analysis showed no demonstrated difference in risk ratio of SAE between patients with CKD (5 trials, 9696 patients; RR 0.95, 95% CI 0.87–1.04, *I*^2^ = 25%) and with unknown history of CKD (13 trials, 36 915 patients; RR 0.96, 95% CI 0.89–1.03, *I*^2^ = 84%) (Supplementary data, Fig. S21).

Subgroup analysis performed on different GLP-1RA drug demonstrated increase in incidence of SAE compared with control intervention with semaglutide (8 trials, 16 380 patients; RR 0.92, 95% CI 0.89–0.95, *I*^2^ = 63%) and albiglutide (1 trial, 4731 patients; RR 0.92, 95% CI 0.85–0.99). Higher incidence of SAE was found in control intervention compared with efpeglenatide (1 trial, 2717 patients; RR 1.21, 95% CI 1.13–1.30). There was no demonstrated difference in incidence of serious adverse outcomes with dulaglutide (2 trials, 5331 patients; RR 0.94, 95% CI 0.88–1.02, *I*^2^ = 10%), liraglutide (3 trials, 6070 patients; RR 0.96, 95% CI 0.90–1.03, *I*^2^ = 16%), lixisenatide (1 trial, 3031 patients; RR 0.93, 95% CI 0.85–1.03) and exenatide (1 trial, 7356 patients; RR 1.02, 95% CI 0.94–1.09) (Supplementary data, Fig. S23).

## DISCUSSION

### Key findings

This systematic review and meta-analysis assessed the effect of GLP1-RAs on renal outcomes in both diabetic and non-diabetic populations. In both patient cohorts, there was a significant reduction in the composite of renal outcomes (defined by renal functional decline, annual decline in eGFR and/or microalbuminuria). Whilst there was no statistically significant reduction in development of kidney failure with GLP1-RAs, this was a relatively rare outcome, and the effect estimate was directionally consistent with our primary outcome. Furthermore, there were significant cardiac and metabolic benefits. These findings add to increasing evidence of the broad effects of GLP1-RAs, extending beyond their role in glycaemic control to renal protection and cardiovascular benefits. We found no significant increase in severe adverse events, acute kidney injury or hypoglycaemia. However, gastrointestinal side effects, a well-reported limitation of GLP1-RAs [28], were more common.

### Relationship to previous studies

GLP1-RAs have been proposed as complementary therapy in CKD due to their potential reno-protective mechanisms. There are many hypotheses on their renal protective effects. For example, improvement of conventional risk factors such as glycaemic control and weight control may indirectly provide renal protective effects. However, GLP1-RAs may have direct protective effects such as decreased oxidative stress and decreased expression of several inflammatory biomarkers [29]. Moreover, GLP1-RAs enhance natriuresis and improve endothelial function, likely due to inhibition of sodium–hydrogen exchanger 3 in renal proximal tubular cells. This increases distal sodium transport to the macula densa and reduces intraglomerular pressure [30].

Whilst there are recommendations including use of GLP1-RAs in the management of patients with diabetes and kidney disease [13], there is limited evidence on the impact of GLP-1RAs on renal outcomes among non-diabetic populations, which are under-represented in clinical trials. Given the comparable efficacy highlighted in our review, this disparity highlights the need for further research in patients with no history of diabetes, particularly those with CKD and high cardiovascular risk. In addition, the lack of reduction in progression to kidney failure warrants further investigation. The reduction in composite renal outcomes and demonstration of renal protection is important; however, patients with advanced CKD were excluded in the majority of these trials, particularly those with eGFR <30 mL/min/1.73 m^2^. Therefore, the renal effects of GLP1-RAs remain unknown in patients with advanced CKD and future long-term studies should focused on significant renal endpoints are warranted.

Prior systematics reviews have been conducted reviewing effects of GLP1-RAs on renal outcomes [31, 32]. Unlike these studies, we primarily focused on renal outcomes and did not review cardiovascular composite outcomes. We also reviewed varying degrees of CKD, resulting in inclusion of more trials and further subgroup analyses based on the degree of CKD. However, similar to their findings, we noted reduction in both composite kidney outcomes and eGFR decline. Furthermore, we had also highlighted discrepancies in patient populations with more studies focusing on patients with diabetes, exemplifying the need for further studies in patients without diabetes.

### Implications of study findings

Our findings imply that GLP1-RAs, initially used for people with diabetes and obesity, are associated with better renal and cardiovascular outcomes as well as survival benefits. Moreover, this cardiovascular and renal benefits exist in the non-diabetic population. These findings suggest that GLP1-RAs should be considered in patients with proteinuric CKD following renin–angiotensin–aldosterone system blockade and sodium-glucose cotransporter 2 inhibitors (SGLT2i).

### Strengths and limitations

To our knowledge, this is the most comprehensive systematic review to assess the effectiveness of GLP1-RAs on renal outcomes. First, we assessed more RCTs enrolled in our meta-analysis with the inclusion of 19 trials, including the SMART and FLOW trials, which were not included in previous systematic reviews [31, 32]. Second, we explored various renal outcomes including a composite of key renal outcomes. Third, our study focused on different subgroup analyses with exploration in patients with and without diabetes, patients with and without CKD, and individual GLP1-RAs medications.

We acknowledge some limitations. First, the inclusion of trials with varying populations and endpoints introduces heterogeneity; however, most outcomes demonstrated low to moderate statistical heterogeneity. Second, the relatively short follow-up duration in some studies may have underestimated the long-term impact of GLP1-RAs on renal and cardiovascular outcomes. Third, there is variability of our definition of CKD across included studies in our subgroup analyses, affecting comparability of results across patient populations and introducing misclassification bias. Finally, similar to previous analyses [32], the lack of data on certain patient subgroups including patients with advanced CKD, patients with no history of diabetes and those of low cardiovascular risk limits the generalizability of our findings.

## CONCLUSION

Our findings suggest use of GLP1-RAs have significant renal, cardiovascular and metabolic benefits in patients with diabetes and reduce the risk of composite renal outcomes, decline in renal function, and development of microalbuminuria. Our findings also suggest comparable efficacy between patients with and without diabetes; however, the discrepancy in the amount of data highlights the need for further research in patients with no history of diabetes, similar to those conducted for SGLT2i drugs.

## Supplementary Material

gfaf193_Supplemental_File

## Data Availability

The data underlying this article are available in the article and in its online supplementary material.
