# Supplementary material for: The effect of GLP-1 receptor agonists on renal outcomes: a systematic review and meta-analysis
Source: Nephrol Dial Transplant. 2025 Sep 22;41(4):681–91. doi: 10.1093/ndt/gfaf193 (PMC13037467; doi:10.1093/ndt/gfaf193)

Supplementary Materials

Title: The effect of GLP-1 receptor agonists on renal outcomes: A systematic review and meta-analysis

Table of Contents

Supplementary Table S1. Search strategy

Supplementary Figure S1. Risk of bias graph – quality of the included trials

Supplementary Figure S2. Risk of bias summary for composite kidney outcomes

Supplementary Figure S3. Risk of bias summary for kidney failure

Supplementary Figure S4: Risk of bias summary for substantial loss of kidney failure

Supplementary Figure S5: Risk of bias summary for annual change in estimated glomerular filtration rate

Supplementary Figure S6: Risk of bias summary for change in albuminuria

Supplementary Figure S7: Risk of bias summary for incidence macroalbuminuria

Supplementary Figure S8: Risk of bias summary for major adverse cardiovascular outcomes

Supplementary Figure S9: Risk of bias summary for all-cause mortality

Supplementary Figure S10: Risk of bias summary for change in glycated haemoglobin

Supplementary Figure S11: Risk of bias summary for change in body weight

Supplementary Figure S12: Risk of bias summary for serious adverse events

Supplementary Figure S13: Risk of bias summary for gastrointestinal adverse events

Supplementary Figure S14: Risk of bias summary for severe hypoglycaemia

Supplementary Figure S15: Risk of bias summary for acute kidney injury

Supplementary Figure S16: The effect of glucagon-like peptide 1 receptor agonists on glycated haemoglobin and body weight

Supplementary Figure S17: Adverse outcomes

Supplementary Figure S18: The effect of GLP-1 RAs on primary outcomes according to diabetic status

Supplementary Figure S19: Differences in adverse events related to GLP1-RAs based on diabetic status

Supplementary Figure S20: The effect of GLP1-RAs on primary outcomes according to pre-existing chronic kidney disease

Supplementary Figure S21: Differences in adverse events related to GLP-1RAs based on chronic kidney disease status

Supplementary Figure S22: The effect of individual GLP1-RAs on primary outcomes

Supplementary Figure S23: Differences in adverse events with individual GLP1-RAs

Supplementary Table S1. Search strategy

Medline

1 exp Diabetes Mellitus/ or diabet*.ti,ab.

2 exp Obesity/ or obes*.ti,ab.

3. exp Renal Insufficiency, Chronic/ or ("kidney failure" or "kidney disease" or CKD).ti,ab.
4. 1 or 2 or 3
5. exp Glucagon-Like Peptide 1/ or Glucagon-Like Peptide-1 Receptor agonist/ or "GLP 1".ti,ab.

6. exenatide/ or lixisenatide/ or semaglutide/ or liraglutide/ or exenatide/ or dulaglutide/ or taspoglutide/ or albiglutide/ or Efpeglenatide/

7. (glucagon like peptide* or GLP-1).tw

8. 5 or 6 or 7

9. 4 and 8

10. ((kidney or renal) adj2 (improve* or outcome* or function or impair*)).ti,ab.

11. randomized controlled trial.pt.

12. controlled clinical trial.pt.

13. randomised.ab.

14. placebo.ab.

15. 11 or 12 or 13 or 14

16. 9 and 10 and 15

Embase

1. 'diabetes mellitus'/exp OR 'diabetes mellitus' OR diabet*:ti OR diabet*:ab
2. 'obesity'/exp OR 'obesity' OR 'obes*':ti OR 'obes*':ab
3. 'renal insufficiency, chronic'/exp OR 'renal insufficiency, chronic' OR 'kidney failure':ti OR 'kidney disease':ti OR ckd:ti OR 'kidney failure':ab OR 'kidney disease':ab
4. 1 or 2 or 3
5. Glucagon-Like Peptide 1/exp
6. (Glucagon-Like Peptide 1 or GLP-1):TI,AB

7. (exenatide or liraglutide or albiglutide or taspoglutide or lixisenatide) :TI,AB

8. 5 or 6 or 7

9. (((kidney OR renal) NEAR/2 (improve* OR outcome* OR function OR impair*)):ti) OR (((kidney OR renal) NEAR/2 (improve* OR outcome* OR function OR impair*)):ab)

10. randomized controlled trial/exp

11. (randomised or randomized) :TI,AB

12. 10 or 11

13. 4 and 8 and 9 and 12

Cochrane Central

#1 MeSH descriptor Diabetes Mellitus explode all trees

#2 (diabet*):ti,ab,kw (Word variations have been searched)

#3 #1 or #2

#4 MeSH descriptor Obesity explode all trees

#5 (obes*):ti,ab,kw (Word variations have been searched)

#6 #4 or #5

#7 MeSH descriptor:[Renal Insufficiency, Chronic] explode all trees

#8 ("kidney failure" or "kidney disease" or CKD):ti,ab,kw (Word variations have been searched)

#9 #7 or #8

#10 #3 or #6 or #9

#11 MESH DESCRIPTOR Glucagon-Like Peptide 1 EXPLODE ALL TREES WITH QUALIFIERS AA,AG

#12 ("exenatide" or " lixisenatide" or "semaglutide" or "liraglutide" or "dulaglutide" or "taspoglutide" or "albiglutide" or "Efpeglenatide" or “teduglutide” or “elsiglutide”):ti,ab,kw

#13 ((glucagon like peptide* or GLP 1 or GLP1) ADJ3 (analog* or agonist*)):ti,ab,kw

#14 #11 or #12 or #13

#15 #10 and #14

#16 ((kidney OR renal) NEAR/2 (improve* OR outcome* OR function OR impair*)):ti,ab,kw (Word variations have been searched)

#17 #15 and #16

Supplementary Figure S1. Risk of bias graph – quality of the included trials

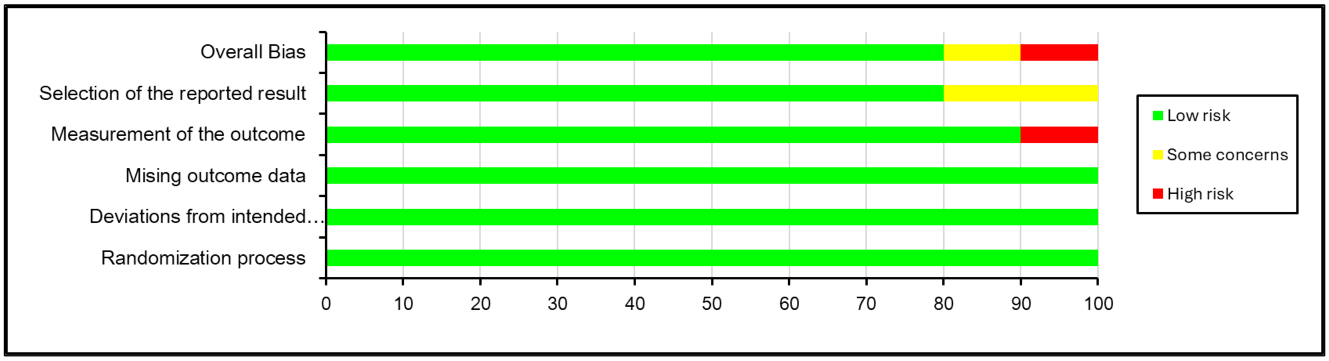

Supplementary Figure S2. Risk of bias summary for composite kidney outcomes

Composite kidney outcomes as defined as combined renal outcomes including kidney dysfunction in each individual study.

Study Name	Intervention	Comparator	Outcome	D1	D2	D3	D4	D5	Overall
AWARD7	Dulaglutide	Insulin Glargine	Composite renal outcom	+	+	+	-	!	-
EXCEL	Exenatide	Placebo	Composite renal outcom	+	+	+	+	+	+
SUSTAIN6	Semaglutide	Placebo	Composite renal outcom	+	+	+	+	+	+
REWIND	Dulaglutide	Placebo	Composite renal outcom	+	+	+	+	+	+
AMPLITUDE-O	Efpeglenatide	Placebo	Composite renal outcom	+	+	+	+	+	+
SURPASS4	Tirzepatide	Insulin glargine	Composite renal outcom	+	+	+	+	!	!
LEADER	Liraglutide	Placebo	Composite renal outcom	+	+	+	+	+	+
GRADE	Liraglutide	Glargine, Glimepiride, or Sitagliptin	Composite renal outcom	+	+	+	+	+	+
FLOW	Semaglutide	Placebo	Composite renal outcom	+	+	+	+	+	+
SELECT	Semaglutide	Placebo	Composite renal outcom	+	+	+	+	+	+

+ Low risk
! Some concerns
- High risk

D1 Randomisation process
D2 Deviations from the intended interventions
D3 Missing outcome data
D4 Measurement of the outcome
D5 Selection of the reported result

Supplementary Figure S3. Risk of bias summary for kidney failure

Study Name	Intervention	Comparator	Outcome	D1	D2	D3	D4	D5	Overall
EXCEL	Exenatide	Placebo	Kidney failure	+	+	+	+	+	+
SUSTAIN6	Semaglutide	Placebo	Kidney failure	+	+	+	+	!	!
REWIND	Dulaglutide	Placebo	Kidney failure	+	+	+	+	+	+
LEADER	Liraglutide	Placebo	Kidney failure	+	+	+	+	+	+
FLOW	Semaglutide	Placebo	Kidney failure	+	+	+	+	+	+
SELECT	Semaglutide	Placebo	Kidney failure	+	+	+	+	+	+

+ Low risk
! Some concerns
- High risk

D1 Randomisation process
D2 Deviations from the intended interventions
D3 Missing outcome data
D4 Measurement of the outcome
D5 Selection of the reported result

Supplementary Figure 4: Risk of bias summary for substantial loss of kidney failure

Study Name	Intervention	Comparator	Outcome	D1	D2	D3	D4	D5	Overall
EXCEL	Exenatide	Placebo	Substantial loss of kidney function	+	+	+	+	+	+
SUSTAIN6	Semaglutide	Placebo	Substantial loss of kidney function	+	+	+	+	!	!
PIONEER6	Semaglutide	Placebo	Substantial loss of kidney function	+	+	+	+	!	!
REWIND	Dulaglutide	Placebo	Substantial loss of kidney function	+	+	+	+	+	+
LEADER	Liraglutide	Placebo	Substantial loss of kidney function	+	+	+	+	+	+
ELIXA	Lixisenatide	Placebo	Substantial loss of kidney function	+	+	+	+	!	!
GRADE	Liraglutide	Glargine, Glimepiride, or Sitagliptin	Substantial loss of kidney function	+	+	+	+	+	+
FLOW	Semaglutide	Placebo	Substantial loss of kidney function	+	+	+	+	+	+
SELECT	Semaglutide	Placebo	Substantial loss of kidney function	+	+	+	+	+	+

+ Low risk

! Some concerns

- High risk

D1 Randomisation process

D2 Deviations from the intended interventions

D3 Missing outcome data

D4 Measurement of the outcome

D5 Selection of the reported result

Supplementary Figure 5: Risk of bias summary for annual change in estimated glomerular filtration rate

Abbreviations: eGFR, estimated glomerular filtration rate

Study Name	Intervention	Comparator	Outcome	D1	D2	D3	D4	D5	Overall
AWARD7	Dulaglutide	Insulin Glargine	Annual eGFR slope	+	+	+	+	+	+
EXCEL	Exenatide	Placebo	Annual eGFR slope	+	+	+	+	+	+
SUSTAIN6	Semaglutide	Placebo	Annual eGFR slope	+	+	+	+	!	!
PIONEER6	Semaglutide	Placebo	Annual eGFR slope	+	+	+	+	!	!
LIRA-RENAL	Liraglutide	Placebo	Annual eGFR slope	+	+	+	+	+	+
HARMONY	Albiglutide	Placebo	Annual eGFR slope	+	+	+	+	+	+
REWIND	Dulaglutide	Placebo	Annual eGFR slope	+	+	+	+	+	+
SURPASS4	Tirzepatide	Insulin glargine	Annual eGFR slope	+	+	+	+	!	!
STEP1	Semaglutide	Placebo	Annual eGFR slope	+	+	+	+	!	!
STEP2	Semaglutide	Placebo	Annual eGFR slope	+	+	+	+	!	!
STEP3	Semaglutide	Placebo	Annual eGFR slope	+	+	+	+	!	!
ChiCTR-1701082	Exenatide	Insulin Glargine	Annual eGFR slope	+	+	+	+	+	+
LEADER	Liraglutide	Placebo	Annual eGFR slope	+	+	+	+	+	+
ELIXA	Lixisenatide	Placebo	Annual eGFR slope	+	+	+	+	!	!
GRADE	Liraglutide	Glargine, Glimepiride, or Sitagliptin	Annual eGFR slope	+	+	+	+	+	+
FLOW	Semaglutide	Placebo	Annual eGFR slope	+	+	+	+	+	+
SELECT	Semaglutide	Placebo	Annual eGFR slope	+	+	+	+	!	!
SMART	Semaglutide	Placebo	Annual eGFR slope	+	+	+	+	+	+

+ Low risk
! Some concerns
- High risk

D1 Randomisation process
D2 Deviations from the intended interventions
D3 Missing outcome data
D4 Measurement of the outcome
D5 Selection of the reported result

Supplementary Figure 6: Risk of bias summary for change in albuminuria

Study Name	Intervention	Comparator	Outcome	D1	D2	D3	D4	D5	Overall
AWARD7	Dulaglutide	Insulin Glargine	Change in proteinuria	+	+	+	+	+	+
SUSTAIN6	Semaglutide	Placebo	Change in proteinuria	+	+	+	+	!	!
LIRA-RENAL	Liraglutide	Placebo	Change in proteinuria	+	+	+	+	+	+
REWIND	Dulaglutide	Placebo	Change in proteinuria	+	+	+	+	+	+
SURPASS4	Tirzepatide	Insulin glargine	Change in proteinuria	+	+	+	+	!	!
STEP2	Semaglutide	Placebo	Change in proteinuria	+	+	+	+	!	!
LEADER	Liraglutide	Placebo	Change in proteinuria	+	+	+	+	+	+
ELIXA	Lixisenatide	Placebo	Change in proteinuria	+	+	+	+	+	+
GRADE	Liraglutide	Glargine, Glimepiride, or Sitagliptin	Change in proteinuria	+	+	+	+	+	+
FLOW	Semaglutide	Placebo	Change in proteinuria	+	+	+	+	+	+
SELECT	Semaglutide	Placebo	Change in proteinuria	+	+	+	+	!	!
SMART	Semaglutide	Placebo	Change in proteinuria	+	+	+	+	+	+

+ Low risk
! Some concerns
- High risk

D1 Randomisation process
D2 Deviations from the intended interventions
D3 Missing outcome data
D4 Measurement of the outcome
D5 Selection of the reported result

Supplementary Figure 7: Risk of bias summary for incidence macroalbuminuria

Study Name	Intervention	Comparator	Outcome	D1	D2	D3	D4	D5	Overall
EXCEL	Exenatide	Placebo	Incident macroalbuminuria	+	+	+	+	+	+
SUSTAIN6	Semaglutide	Placebo	Incident macroalbuminuria	+	+	+	+	!	!
REWIND	Dulaglutide	Placebo	Incident macroalbuminuria	+	+	+	+	+	+
AMPLITUDE-O	Efpeglenatide	Placebo	Incident macroalbuminuria	+	+	+	+	+	+
SURPASS4	Tirzepatide	Insulin glargine	Incident macroalbuminuria	+	+	+	+	!	!
LEADER	Liraglutide	Placebo	Incident macroalbuminuria	+	+	+	+	+	+
ELIXA	Lixisenatide	Placebo	Incident macroalbuminuria	+	+	+	+	+	+
GRADE	Liraglutide	Glargine, Glimepiride, or Sitagliptin	Incident macroalbuminuria	+	+	+	+	+	+
SELECT	Semaglutide	Placebo	Incident macroalbuminuria	+	+	+	+	+	+

+ Low risk
! Some concerns
- High risk

D1 Randomisation process
D2 Deviations from the intended interventions
D3 Missing outcome data
D4 Measurement of the outcome
D5 Selection of the reported result

Supplementary Figure 8: Risk of bias summary for major adverse cardiovascular outcomes

Abbreviations: MACE, major adverse cardiovascular outcomes

Study Name	Intervention	Comparator	Outcome	D1	D2	D3	D4	D5	Overall
EXCEL	Exenatide	Placebo	MACE	+	+	+	+	+	+
SUSTAIN6	Semaglutide	Placebo	MACE	+	+	+	+	+	+
PIONEER6	Semaglutide	Placebo	MACE	+	+	+	+	+	+
HARMONY	Albiglutide	Placebo	MACE	+	+	+	+	+	+
REWIND	Dulaglutide	Placebo	MACE	+	+	+	+	+	+
AMPLITUDE-O	Efpeglenatide	Placebo	MACE	+	+	+	+	+	+
SURPASS4	Tirzepatide	Insulin glargine	MACE	+	+	+	+	+	+
STEP1	Semaglutide	Placebo	MACE	+	+	+	+	+	+
STEP2	Semaglutide	Placebo	MACE	+	+	+	+	+	+
STEP3	Semaglutide	Placebo	MACE	+	+	+	+	+	+
LEADER	Liraglutide	Placebo	MACE	+	+	+	+	+	+
ELIXA	Lixisenatide	Placebo	MACE	+	+	+	+	+	+
GRADE	Liraglutide	Glargine, Glimepiride, or Sitagliptin	MACE	+	+	+	+	+	+
FLOW	Semaglutide	Placebo	MACE	+	+	+	+	+	+
SELECT	Semaglutide	Placebo	MACE	+	+	+	+	+	+

+ Low risk
! Some concerns
- High risk

D1 Randomisation process
D2 Deviations from the intended interventions
D3 Missing outcome data
D4 Measurement of the outcome
D5 Selection of the reported result

Supplementary Figure 9: Risk of bias summary for all-cause mortality

Study Name	Intervention	Comparator	Outcome	D1	D2	D3	D4	D5	Overall
AWARD7	Dulaglutide	Insulin Glargine	All-cause mortality	+	+	+	+	+	+
EXCEL	Exenatide	Placebo	All-cause mortality	+	+	+	+	+	+
SUSTAIN6	Semaglutide	Placebo	All-cause mortality	+	+	+	+	+	+
PIONEER6	Semaglutide	Placebo	All-cause mortality	+	+	+	+	+	+
LIRA-RENAL	Liraglutide	Placebo	All-cause mortality	+	+	+	+	+	+
HARMONY	Albiglutide	Placebo	All-cause mortality	+	+	+	+	+	+
REWIND	Dulaglutide	Placebo	All-cause mortality	+	+	+	+	+	+
AMPLITUDE-O	Efpeglenatide	Placebo	All-cause mortality	+	+	+	+	+	+
SURPASS4	Tirzepatide	Insulin glargine	All-cause mortality	+	+	+	+	+	+
STEP1	Semaglutide	Placebo	All-cause mortality	+	+	+	+	+	+
STEP2	Semaglutide	Placebo	All-cause mortality	+	+	+	+	+	+
LEADER	Liraglutide	Placebo	All-cause mortality	+	+	+	+	+	+
ELIXA	Lixisenatide	Placebo	All-cause mortality	+	+	+	+	+	+
GRADE	Liraglutide	Glargine, Glimepiride, or Sitagliptin	All-cause mortality	+	+	+	+	+	+
FLOW	Semaglutide	Placebo	All-cause mortality	+	+	+	+	+	+
SELECT	Semaglutide	Placebo	All-cause mortality	+	+	+	+	+	+

+ Low risk
! Some concerns
- High risk

D1 Randomisation process
D2 Deviations from the intended interventions
D3 Missing outcome data
D4 Measurement of the outcome
D5 Selection of the reported result

Supplementary Figure 10: Risk of bias summary for change in glycated haemoglobin

Abbreviations: HbA1c, glycated haemoglobin

Study Name	Intervention	Comparator	Outcome	D1	D2	D3	D4	D5	Overall
AWARD7	Dulaglutide	Insulin Glargine	Change in HbA1c	+	+	+	+	+	+
EXCEL	Exenatide	Placebo	Change in HbA1c	+	+	+	+	+	+
SUSTAIN6	Semaglutide	Placebo	Change in HbA1c	+	+	+	+	+	+
PIONEER6	Semaglutide	Placebo	Change in HbA1c	+	+	+	+	+	+
LIRA-RENAL	Liraglutide	Placebo	Change in HbA1c	+	+	+	+	+	+
HARMONY	Albiglutide	Placebo	Change in HbA1c	+	+	+	+	+	+
REWIND	Dulaglutide	Placebo	Change in HbA1c	+	+	+	+	+	+
SURPASS4	Tirzepatide	Insulin glargine	Change in HbA1c	+	+	+	+	+	+
STEP1	Semaglutide	Placebo	Change in HbA1c	+	+	+	+	+	+
STEP2	Semaglutide	Placebo	Change in HbA1c	+	+	+	+	+	+
STEP3	Semaglutide	Placebo	Change in HbA1c	+	+	+	+	+	+
ChiCTR-1701082	Exenatide	Insulin Glargine	Change in HbA1c	+	+	+	+	+	+
LEADER	Liraglutide	Placebo	Change in HbA1c	+	+	+	+	+	+
FLOW	Semaglutide	Placebo	Change in HbA1c	+	+	+	+	+	+

+ Low risk
! Some concerns
- High risk

D1 Randomisation process
D2 Deviations from the intended interventions
D3 Missing outcome data
D4 Measurement of the outcome
D5 Selection of the reported result

Supplementary Figure 11: Risk of bias summary for change in body weight

Abbreviations: BW, body weight

Study Name	Intervention	Comparator	Outcome	D1	D2	D3	D4	D5	Overall
AWARD7	Dulaglutide	Insulin Glargine	Change in BW	+	+	+	+	+	+
EXCEL	Exenatide	Placebo	Change in BW	+	+	+	+	+	+
SUSTAIN6	Semaglutide	Placebo	Change in BW	+	+	+	+	+	+
PIONEER6	Semaglutide	Placebo	Change in BW	+	+	+	+	+	+
LIRA-RENAL	Liraglutide	Placebo	Change in BW	+	+	+	+	+	+
HARMONY	Albiglutide	Placebo	Change in BW	+	+	+	+	+	+
REWIND	Dulaglutide	Placebo	Change in BW	+	+	+	+	+	+
SURPASS4	Tirzepatide	Insulin glargine	Change in BW	+	+	+	+	+	+
STEP1	Semaglutide	Placebo	Change in BW	+	+	+	+	+	+
STEP2	Semaglutide	Placebo	Change in BW	+	+	+	+	+	+
STEP3	Semaglutide	Placebo	Change in BW	+	+	+	+	+	+
ChiCTR-1701082	Exenatide	Insulin Glargine	Change in BW	+	+	+	+	+	+
LEADER	Liraglutide	Placebo	Change in BW	+	+	+	+	+	+
FLOW	Semaglutide	Placebo	Change in BW	+	+	+	+	+	+
SMART	Semaglutide	Placebo	Change in BW	+	+	+	+	+	+

\+ Low risk
! Some concerns
\- High risk

D1 Randomisation process
D2 Deviations from the intended interventions
D3 Missing outcome data
D4 Measurement of the outcome
D5 Selection of the reported result

Supplementary Figure 12: Risk of bias summary for serious adverse events

Abbreviations: AE, adverse events

Study Name	Intervention	Comparator	Outcome	D1	D2	D3	D4	D5	Overall
AWARD7	Dulaglutide	Insulin Glargine	Serious AE	+	+	+	-	+	-
EXCEL	Exenatide	Placebo	Serious AE	+	+	+	+	+	+
SUSTAIN6	Semaglutide	Placebo	Serious AE	+	+	+	+	+	+
PIONEER6	Semaglutide	Placebo	Serious AE	+	+	+	+	+	+
LIRA-RENAL	Liraglutide	Placebo	Serious AE	+	+	+	+	+	+
HARMONY	Albiglutide	Placebo	Serious AE	+	+	+	+	+	+
REWIND	Dulaglutide	Placebo	Serious AE	+	+	+	+	+	+
AMPLITUDE-O	Efpeglenatide	Placebo	Serious AE	+	+	+	+	+	+
SURPASS4	Tirzepatide	Insulin glargine	Serious AE	+	+	+	-	+	-
STEP1	Semaglutide	Placebo	Serious AE	+	+	+	+	+	+
STEP2	Semaglutide	Placebo	Serious AE	+	+	+	+	+	+
STEP3	Semaglutide	Placebo	Serious AE	+	+	+	+	+	+
LEADER	Liraglutide	Placebo	Serious AE	+	+	+	+	+	+
ELIXA	Lixisenatide	Placebo	Serious AE	+	+	+	+	+	+
GRADE	Liraglutide	Glargine, Glimepiride, or Sitagliptin	Serious AE	+	+	+	-	+	-
FLOW	Semaglutide	Placebo	Serious AE	+	+	+	+	+	+
SELECT	Semaglutide	Placebo	Serious AE	+	+	+	+	+	+
SMART	Semaglutide	Placebo	Serious AE	+	+	+	+	+	+

+	Low risk
!	Some concerns
-	High risk

D1	Randomisation process
D2	Deviations from the intended interventions
D3	Missing outcome data
D4	Measurement of the outcome
D5	Selection of the reported result

Supplementary Figure 13: Risk of bias summary for gastrointestinal adverse events

Abbreviations: AE, adverse events

Study Name	Intervention	Comparator	Outcome	D1	D2	D3	D4	D5	Overall
AWARD7	Dulaglutide	Insulin Glargine	Gastro Intestinal AE	+	+	+	-	+	-
EXCEL	Exenatide	Placebo	Gastro Intestinal AE	+	+	+	+	+	+
SUSTAIN6	Semaglutide	Placebo	Gastro Intestinal AE	+	+	+	+	+	+
PIONEER6	Semaglutide	Placebo	Gastro Intestinal AE	+	+	+	+	+	+
LIRA-RENAL	Liraglutide	Placebo	Gastro Intestinal AE	+	+	+	+	+	+
HARMONY	Albiglutide	Placebo	Gastro Intestinal AE	+	+	+	+	+	+
REWIND	Dulaglutide	Placebo	Gastro Intestinal AE	+	+	+	+	+	+
AMPLITUDE-O	Efpeglenatide	Placebo	Gastro Intestinal AE	+	+	+	+	+	+
SURPASS4	Tirzepatide	Insulin glargine	Gastro Intestinal AE	+	+	+	-	+	-
STEP1	Semaglutide	Placebo	Gastro Intestinal AE	+	+	+	+	+	+
STEP2	Semaglutide	Placebo	Gastro Intestinal AE	+	+	+	+	+	+
STEP3	Semaglutide	Placebo	Gastro Intestinal AE	+	+	+	+	+	+
LEADER	Liraglutide	Placebo	Gastro Intestinal AE	+	+	+	+	+	+
ELIXA	Lixisenatide	Placebo	Gastro Intestinal AE	+	+	+	+	+	+
GRADE	Liraglutide	Glargine, Glimepiride, or Sitagliptin	Gastro Intestinal AE	+	+	+	-	+	-
FLOW	Semaglutide	Placebo	Gastro Intestinal AE	+	+	+	+	+	+
SELECT	Semaglutide	Placebo	Gastro Intestinal AE	+	+	+	+	+	+
SMART	Semaglutide	Placebo	Gastro Intestinal AE	+	+	+	+	+	+

+ Low risk
! Some concerns
- High risk

D1 Randomisation process
D2 Deviations from the intended interventions
D3 Missing outcome data
D4 Measurement of the outcome
D5 Selection of the reported result

Supplementary Figure 14: Risk of bias summary for severe hypoglycaemia

Abbreviations: AE, adverse events

Study Name	Intervention	Comparator	Outcome	D1	D2	D3	D4	D5	Overall
AWARD7	Dulaglutide	Insulin Glargine	Severe hypoglycemic AE	+	+	+	-	+	-
EXCEL	Exenatide	Placebo	Severe hypoglycemic AE	+	+	+	+	+	+
SUSTAIN6	Semaglutide	Placebo	Severe hypoglycemic AE	+	+	+	+	+	+
PIONEER6	Semaglutide	Placebo	Severe hypoglycemic AE	+	+	+	+	+	+
LIRA-RENAL	Liraglutide	Placebo	Severe hypoglycemic AE	+	+	+	+	+	+
HARMONY	Albiglutide	Placebo	Severe hypoglycemic AE	+	+	+	+	+	+
REWIND	Dulaglutide	Placebo	Severe hypoglycemic AE	+	+	+	+	+	+
AMPLITUDE-O	Efpeglenatide	Placebo	Severe hypoglycemic AE	+	+	+	+	+	+
SURPASS4	Tirzepatide	Insulin glargine	Severe hypoglycemic AE	+	+	+	-	+	-
STEP1	Semaglutide	Placebo	Severe hypoglycemic AE	+	+	+	+	+	+
STEP2	Semaglutide	Placebo	Severe hypoglycemic AE	+	+	+	+	+	+
LEADER	Liraglutide	Placebo	Severe hypoglycemic AE	+	+	+	+	+	+
ELIXA	Lixisenatide	Placebo	Severe hypoglycemic AE	+	+	+	+	+	+
GRADE	Liraglutide	Glargine, Glimepiride, or Sitagliptin	Severe hypoglycemic AE	+	+	+	-	+	-
FLOW	Semaglutide	Placebo	Severe hypoglycemic AE	+	+	+	+	+	+

+	Low risk
!	Some concerns
-	High risk
D1	Randomisation process
D2	Deviations from the intended interventions
D3	Missing outcome data
D4	Measurement of the outcome
D5	Selection of the reported result

Supplementary Figure 15: Risk of bias summary for acute kidney injury

Abbreviations: AKI, acute kidney injury

Study Name	Intervention	Comparator	Outcome	D1	D2	D3	D4	D5	Overall
SUSTAIN6	Semaglutide	Placebo	AKI	+	+	+	+	+	+
PIONEER6	Semaglutide	Placebo	AKI	+	+	+	+	+	+
LIRA-RENAL	Liraglutide	Placebo	AKI	+	+	+	+	+	+
HARMONY	Albiglutide	Placebo	AKI	+	+	+	+	+	+
REWIND	Dulaglutide	Placebo	AKI	+	+	+	+	+	+
AMPLITUDE-O	Efpeglenatide	Placebo	AKI	+	+	+	+	+	+
STEP1	Semaglutide	Placebo	AKI	+	+	+	+	+	+
STEP2	Semaglutide	Placebo	AKI	+	+	+	+	+	+
LEADER	Liraglutide	Placebo	AKI	+	+	+	+	+	+
ELIXA	Lixisenatide	Placebo	AKI	+	+	+	+	+	+
FLOW	Semaglutide	Placebo	AKI	+	+	+	+	+	+
SELECT	Semaglutide	Placebo	AKI	+	+	+	+	+	+

\+ Low risk
! Some concerns
\- High risk

D1 Randomisation process
D2 Deviations from the intended interventions
D3 Missing outcome data
D4 Measurement of the outcome
D5 Selection of the reported result

Supplementary Figure 16: The effect of glucagon-like peptide 1 receptor agonists on glycated haemoglobin and body weight

Glycated haemoglobin (A) and body weight (B)

Abbreviations: MD, mean differences; CI, confidence intervals; GLP1-RA, glucagon-like peptide 1 receptor agonists; HbA1c, glycated haemoglobin; BW, body weight

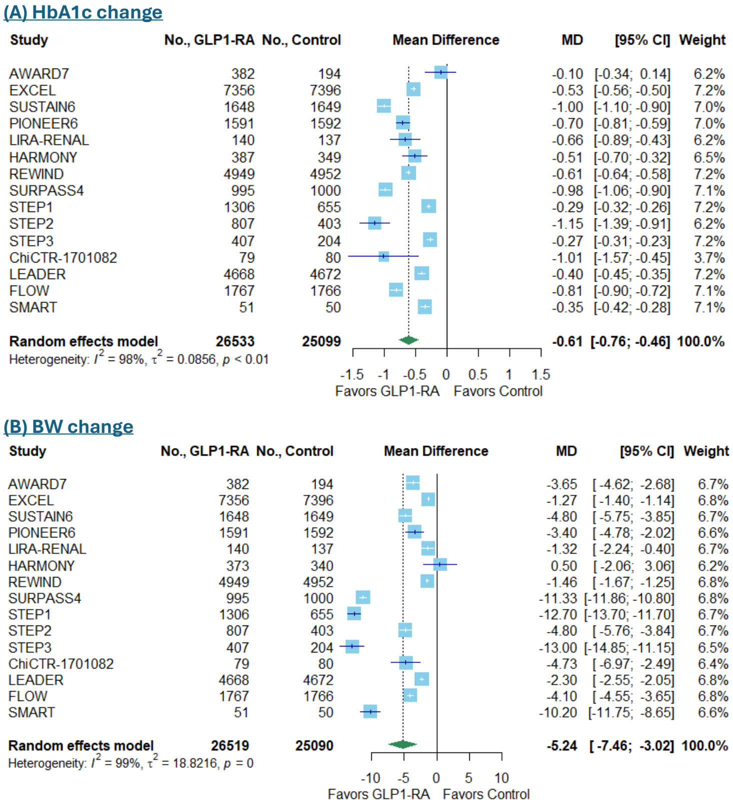

Supplementary Figure 17: Adverse outcomes

Adverse outcomes are as follows: serious adverse events (A), gastrointestinal effects (B), severe hypoglycaemia (C), acute kidney injury (D)

Abbreviations: RR, risk ratios; CI, confidence intervals; SAE, serious adverse events; GI, gastrointestinal; AKI, acute kidney injury; GLP1-RA, glucagon-like peptide 1 receptor agonists

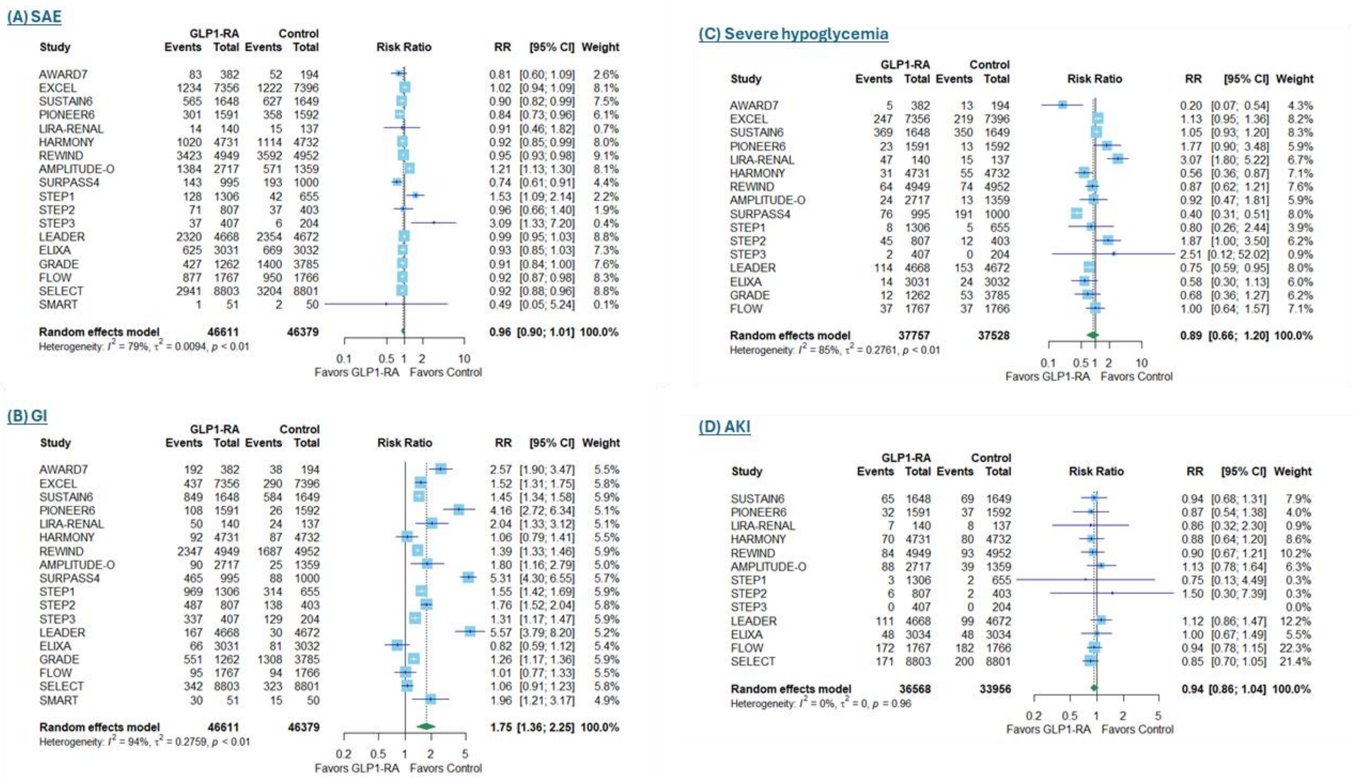

Supplementary Figure S18: The effect of GLP-1 RAs on primary outcomes according to diabetic status

Abbreviations: RR, risk ratios; CI, confidence intervals; CKD, chronic kidney disease; GLP1-RA, glucagon-like peptide 1 receptor agonists

Study	GLP1-RA Events	GLP1-RA Total	Control Events	Control Total	RR	[95% CI]	Weight
**Subgroup = Diabetes**							
AWARD7	26	382	21	194	0.63	[0.36; 1.09]	2.7%
EXCEL	246	6459	266	6466	0.93	[0.78; 1.10]	11.5%
SUSTAIN6	62	1648	100	1649	0.62	[0.46; 0.85]	6.3%
REWIND	848	4949	970	4952	0.87	[0.80; 0.95]	15.6%
AMPLITUDE-O	353	2717	250	1359	0.71	[0.61; 0.82]	12.5%
SURPASS4	63	995	104	1000	0.61	[0.45; 0.82]	6.6%
LEADER	268	4668	337	4672	0.80	[0.68; 0.93]	12.2%
GRADE	152	1262	440	3785	1.04	[0.87; 1.23]	11.3%
FLOW	218	1767	260	1766	0.84	[0.71; 0.99]	11.6%
**Random effects model**		**24847**		**25843**	**0.80**	**[0.72; 0.90]**	**90.2%**
**Subgroup = Non-diabetes**							
SELECT	155	8800	198	8795	0.78	[0.64; 0.96]	9.8%
**Random effects model**		**33647**		**34638**	**0.81**	**[0.73; 0.89]**	**100.0%**

Diabetes subgroup – Heterogeneity: $I^2 = 65\%$, $\tau^2 = 0.0174$, $p < 0.01$; Subgroup effectz = -3.87 ($p < 0.01$)

Overall – Heterogeneity: $I^2 = 61\%$, $\tau^2 = 0.0141$, $p < 0.01$

Test for overall effect: $z = -4.36$ ($p < 0.01$)

Test for subgroup differences: $\chi^2_1 = 0.05$, df = 1 ($p = 0.81$)

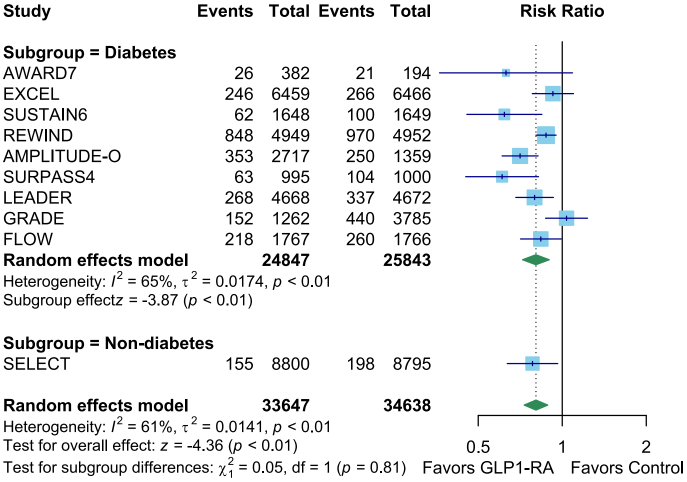

Supplementary Figure S19: Differences in adverse events related to GLP1-RAs based on diabetic status

Abbreviations: RR, risk ratios; CI, confidence intervals; CKD, chronic kidney disease; GLP1-RA, glucagon-like peptide 1 receptor agonists

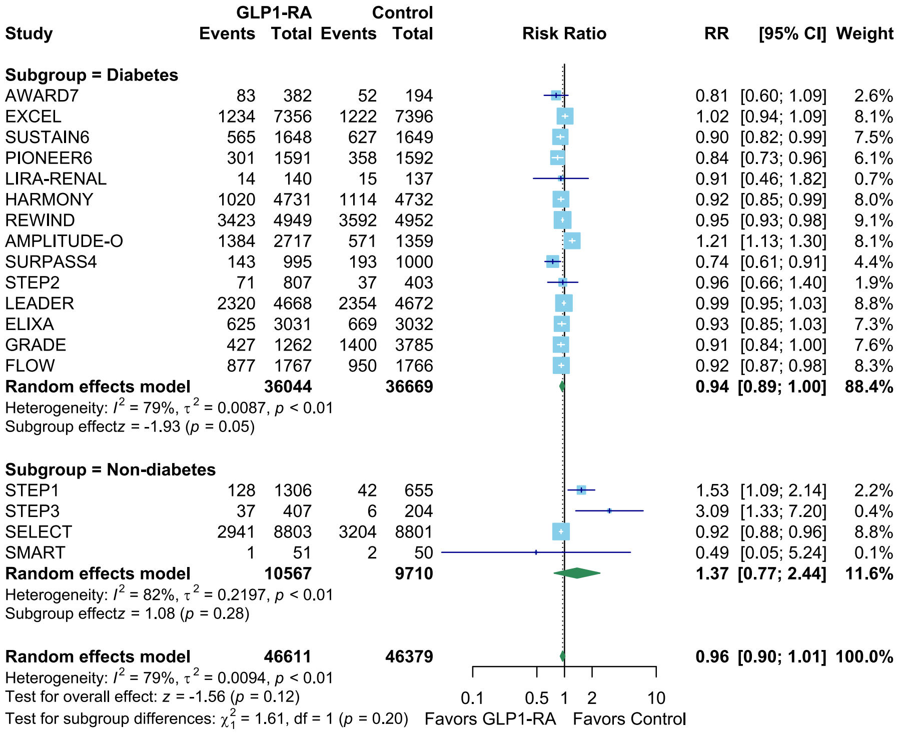

Supplementary Figure S20: The effect of GLP1-RAs on primary outcomes according to pre-existing chronic kidney disease

Abbreviations: RR, risk ratios; CI, confidence intervals; CKD, chronic kidney disease; GLP1-RA, glucagon-like peptide 1 receptor agonists

Study	GLP1-RA Events	GLP1-RA Total	Control Events	Control Total	RR	[95% CI]	Weight
**Subgroup = CKD**							
AWARD7	26	382	21	194	0.63	[0.36; 1.09]	2.1%
EXCEL	246	7326	273	7365	0.91	[0.76; 1.07]	11.3%
SUSTAIN6	60	863	94	838	0.62	[0.45; 0.84]	5.5%
REWIND	219	1081	251	1118	0.90	[0.77; 1.06]	11.8%
AMPLITUDE-O	320	1581	230	794	0.70	[0.60; 0.81]	12.7%
SURPASS4	11	172	24	166	0.44	[0.22; 0.87]	1.4%
LEADER	81	1116	90	1042	0.84	[0.63; 1.12]	6.1%
FLOW	218	1767	260	1766	0.84	[0.71; 0.99]	11.4%
SELECT	75	970	73	938	0.99	[0.73; 1.35]	5.5%
**Random effects model**		**15258**		**14221**	**0.80**	**[0.72; 0.89]**	**67.8%**
Heterogeneity: $I^2 = 50\%$, $\tau^2 = 0.0105$, $p = 0.04$							
Subgroup effectz = -4.03 ($p < 0.01$)							
**Subgroup = Non-CKD**							
SUSTAIN6	2	785	6	811	0.34	[0.07; 1.70]	0.3%
REWIND	610	3734	701	3707	0.86	[0.78; 0.95]	16.0%
AMPLITUDE-O	23	1071	18	537	0.64	[0.35; 1.18]	1.8%
SURPASS4	52	823	80	834	0.66	[0.47; 0.92]	4.9%
LEADER	33	3552	39	3630	0.86	[0.55; 1.37]	2.9%
SELECT	80	7804	125	7834	0.64	[0.49; 0.85]	6.4%
**Random effects model**		**17769**		**17353**	**0.75**	**[0.63; 0.89]**	**32.2%**
Heterogeneity: $I^2 = 33\%$, $\tau^2 = 0.0157$, $p = 0.19$							
Subgroup effectz = -3.27 ($p < 0.01$)							
**Random effects model**		**33027**		**31574**	**0.79**	**[0.73; 0.86]**	**100.0%**
Heterogeneity: $I^2 = 40\%$, $\tau^2 = 0.0092$, $p = 0.05$							
Test for overall effect: $z = -5.42$ ($p < 0.01$)							
Test for subgroup differences: $\chi^2_1 = 0.44$, df = 1 ($p = 0.51$)							

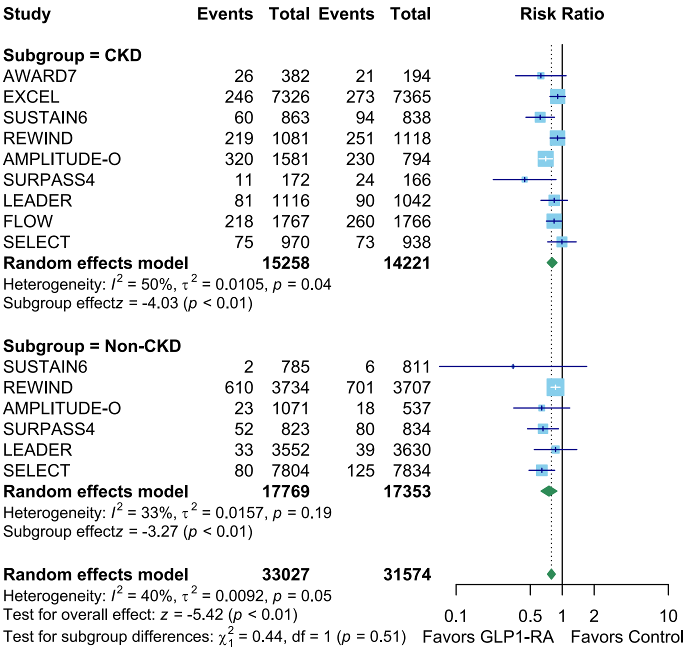

Supplementary Figure S21: Differences in adverse events related to GLP-1RAs based on chronic kidney disease status

Abbreviations: RR, risk ratios; CI, confidence intervals; CKD, chronic kidney disease; GLP1-RA, glucagon-like peptide 1 receptor agonists

*Unknown CKD status defined when trials enrolled both CKD and non-CKD patients but did not report adverse events separately for each group

Study	GLP1-RA Events	GLP1-RA Total	Control Events	Control Total	RR	[95% CI]	Weight
**Subgroup = CKD**							
AWARD7	83	382	52	194	0.81	[0.60; 1.09]	2.6%
EXCEL	1234	7356	1222	7396	1.02	[0.94; 1.09]	8.1%
LIRA-RENAL	14	140	15	137	0.91	[0.46; 1.82]	0.7%
FLOW	877	1767	950	1766	0.92	[0.87; 0.98]	8.3%
SMART	1	51	2	50	0.49	[0.05; 5.24]	0.1%
**Random effects model**		**9696**		**9543**	**0.95**	**[0.87; 1.04]**	**19.7%**
Heterogeneity: $I^2 = 25\%$, $\tau^2 = 0.0032$, $p = 0.25$							
Subgroup effectz = -1.10 (p = 0.27)							
**Subgroup = Unknown CKD status***							
SUSTAIN6	565	1648	627	1649	0.90	[0.82; 0.99]	7.5%
PIONEER6	301	1591	358	1592	0.84	[0.73; 0.96]	6.1%
HARMONY	1020	4731	1114	4732	0.92	[0.85; 0.99]	8.0%
REWIND	3423	4949	3592	4952	0.95	[0.93; 0.98]	9.1%
AMPLITUDE-O	1384	2717	571	1359	1.21	[1.13; 1.30]	8.1%
SURPASS4	143	995	193	1000	0.74	[0.61; 0.91]	4.4%
STEP1	128	1306	42	655	1.53	[1.09; 2.14]	2.2%
STEP2	71	807	37	403	0.96	[0.66; 1.40]	1.9%
STEP3	37	407	6	204	3.09	[1.33; 7.20]	0.4%
LEADER	2320	4668	2354	4672	0.99	[0.95; 1.03]	8.8%
ELIXA	625	3031	669	3032	0.93	[0.85; 1.03]	7.3%
GRADE	427	1262	1400	3785	0.91	[0.84; 1.00]	7.6%
SELECT	2941	8803	3204	8801	0.92	[0.88; 0.96]	8.8%
**Random effects model**		**36915**		**36836**	**0.96**	**[0.89; 1.03]**	**80.3%**
Heterogeneity: $I^2 = 84\%$, $\tau^2 = 0.0133$, $p < 0.01$							
Subgroup effectz = -1.05 (p = 0.29)							
**Random effects model**		**46611**		**46379**	**0.96**	**[0.90; 1.01]**	**100.0%**
Heterogeneity: $I^2 = 79\%$, $\tau^2 = 0.0094$, $p < 0.01$							
Test for overall effect: $z = -1.56$ (p = 0.12)							
Test for subgroup differences: $\chi^2_1 = 0.03$, df = 1 (p = 0.87)							

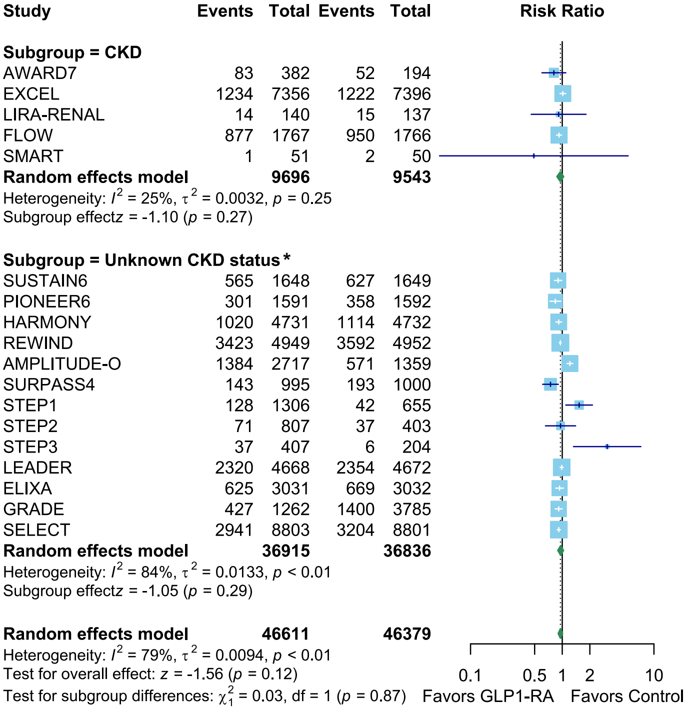

Supplementary Figure S22: The effect of individual GLP1-RAs on primary outcomes

Abbreviations: RR, risk ratios; CI, confidence intervals; GLP1-RA, glucagon-like peptide 1 receptor agonists

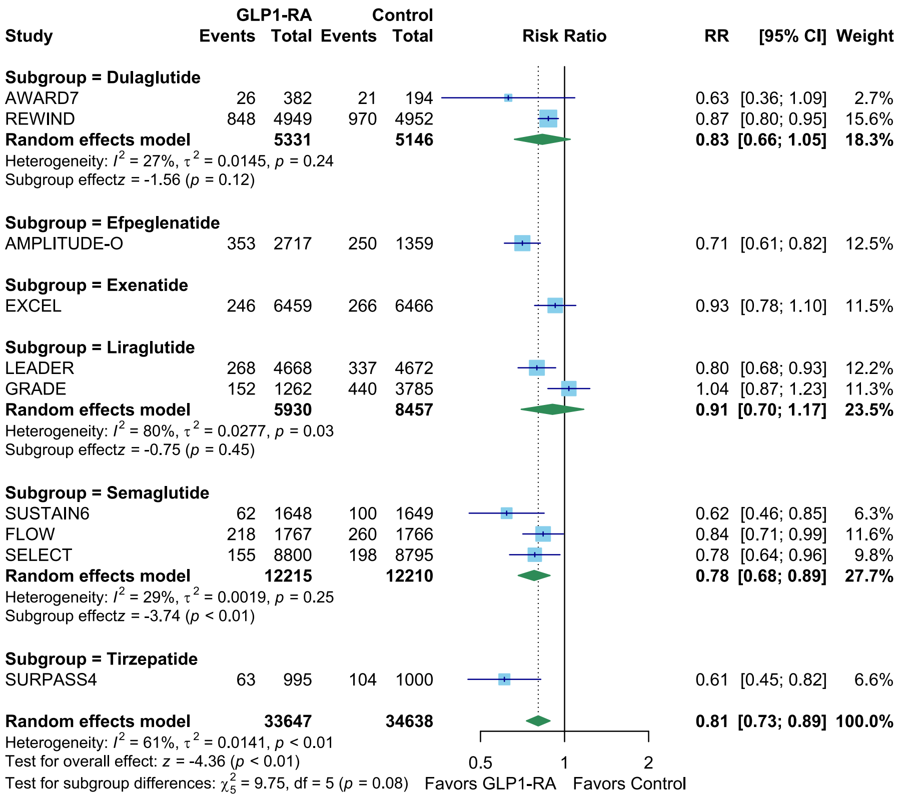

Supplementary Figure S23: Differences in adverse events with individual GLP1-Ras

Abbreviations: RR, risk ratios; CI, confidence intervals; GLP1-RA, glucagon-like peptide 1 receptor agonists

Study	GLP1-RA Events	GLP1-RA Total	Control Events	Control Total	RR	[95% CI]	Weight
**Subgroup = Albiglutide**							
HARMONY	1020	4731	1114	4732	0.92	[0.85; 0.99]	8.0%
**Subgroup = Dulaglutide**							
AWARD7	83	382	52	194	0.81	[0.60; 1.09]	2.6%
REWIND	3423	4949	3592	4952	0.95	[0.93; 0.98]	9.1%
**Random effects model**		**5331**		**5146**	**0.94**	**[0.88; 1.02]**	**11.7%**
Heterogeneity: I^2 = 10%, τ^2 = 0.0013, p = 0.29							
Subgroup effectz = -1.51 (p = 0.13)							
**Subgroup = Efpeglenatide**							
AMPLITUDE-O	1384	2717	571	1359	1.21	[1.13; 1.30]	8.1%
**Subgroup = Exenatide**							
EXCEL	1234	7356	1222	7396	1.02	[0.94; 1.09]	8.1%
**Subgroup = Liraglutide**							
LIRA-RENAL	14	140	15	137	0.91	[0.46; 1.82]	0.7%
LEADER	2320	4668	2354	4672	0.99	[0.95; 1.03]	8.8%
GRADE	427	1262	1400	3785	0.91	[0.84; 1.00]	7.6%
**Random effects model**		**6070**		**8594**	**0.96**	**[0.90; 1.03]**	**17.1%**
Heterogeneity: I^2 = 16%, τ^2 = 0.0016, p = 0.30							
Subgroup effectz = -1.16 (p = 0.25)							
**Subgroup = Lixisenatide**							
ELIXA	625	3031	669	3032	0.93	[0.85; 1.03]	7.3%
**Subgroup = Semaglutide**							
SUSTAIN6	565	1648	627	1649	0.90	[0.82; 0.99]	7.5%
PIONEER6	301	1591	358	1592	0.84	[0.73; 0.96]	6.1%
STEP1	128	1306	42	655	1.53	[1.09; 2.14]	2.2%
STEP2	71	807	37	403	0.96	[0.66; 1.40]	1.9%
STEP3	37	407	6	204	3.09	[1.33; 7.20]	0.4%
FLOW	877	1767	950	1766	0.92	[0.87; 0.98]	8.3%
SELECT	2941	8803	3204	8801	0.92	[0.88; 0.96]	8.8%
SMART	1	51	2	50	0.49	[0.05; 5.24]	0.1%
**Random effects model**		**16380**		**15120**	**0.92**	**[0.89; 0.95]**	**35.3%**
Heterogeneity: I^2 = 63%, τ^2 = < 0.0001, p < 0.01							
Subgroup effectz = -5.38 (p < 0.01)							
**Subgroup = Tirzepatide**							
SURPASS4	143	995	193	1000	0.74	[0.61; 0.91]	4.4%
**Random effects model**		**46611**		**46379**	**0.96**	**[0.90; 1.01]**	**100.0%**
Heterogeneity: I^2 = 79%, τ^2 = 0.0094, p < 0.01							
Test for overall effect: z = -1.56 (p = 0.12)							
Test for subgroup differences: χ^2_7 = 57.98, df = 7 (p < 0.01)							

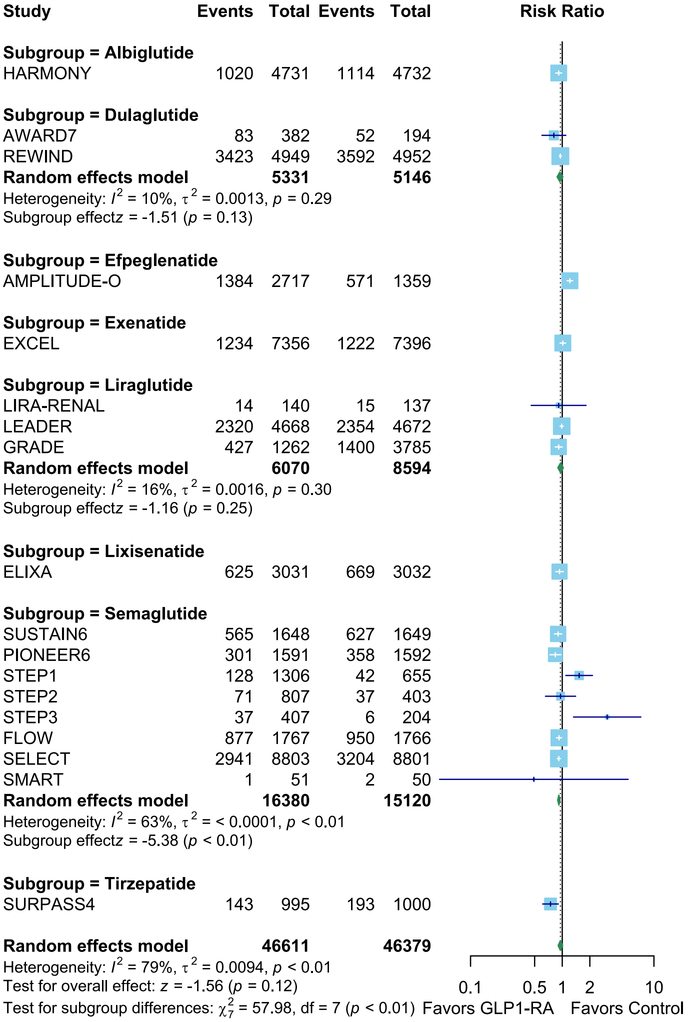

Supplement: gfaf193_Supplemental_File [file gfaf193_supplemental_file.pdf]
